# The molecular size of the extra-membrane domain influences the diffusion of the GPI-anchored VSG on the trypanosome plasma membrane

**DOI:** 10.1038/srep10394

**Published:** 2015-06-11

**Authors:** Andreas J. W. Hartel, Marius Glogger, Gernot Guigas, Nicola G. Jones, Susanne F. Fenz, Matthias Weiss, Markus Engstler

**Affiliations:** 1 Department of Cell and Developmental Biology, Biocenter, University of Würzburg, Würzburg, Germany; 2 Experimental Physics I, University of Bayreuth, Bayreuth, Germany; 3 Department of Electrical Engineering, Bioeelectrical Systems Lab, Columbia University, New York, New York 10027, USA

## Abstract

A plethora of proteins undergo random and passive diffusion in biological membranes. While the contribution of the membrane-embedded domain to diffusion is well established, the potential impact of the extra-membrane protein part has been largely neglected. Here, we show that the molecular length influences the diffusion coefficient of GPI-anchored proteins: smaller proteins diffuse faster than larger ones. The distinct diffusion properties of differently sized membrane proteins are biologically relevant. The variant surface glycoprotein (VSG) of African trypanosomes, for example, is sized for an effective diffusion-driven randomization on the cell surface, a process that is essential for parasite virulence. We propose that the molecular sizes of proteins dominating the cell surfaces of other eukaryotic pathogens may also be related to diffusion-limited functions.

A range of molecular interactions determines the spatio-temporal organisation in biological membranes. The extracellular matrix can bind to surface proteins, and luminal domains can be tethered to the cytoskeleton. Some membrane proteins form aggregates, junctional exclusion can occur at cell-cell interfaces^1^,^2^ and lipid microdomains of variable sizes associate with clusters of membrane proteins^3^. Lateral diffusion is generally considered a background process rather than an organising principle in biological membranes, as it occurs in a random and passive manner.

The diffusion properties of transmembrane proteins are described by the Saffman-Delbrück relation^4^. In this hydrodynamic model the diffusion coefficient of a particle embedded in a membrane and surrounded by a much less viscous fluid is mainly determined by the viscosity and thickness of the bilayer and depends only weakly on the radius of the membrane-spanning domain. A more general model, valid for arbitrary viscosities of the membrane and surrounding medium was provided by Hughes *et al.* in 1981^5^,^6^. To model the lateral diffusion of lipids and proteins in solid supported lipid bilayers, which widely serve as model membranes, Evans and Sackmann extended the continuum model by taking asymmetric boundary conditions and the resulting friction on the membrane into account^7^. The membrane-penetrating part of peripheral membrane proteins follows the Saffman-Delbrück relation, albeit with a few modifications^8^. All the above mentioned models are, however, derived on the assumption of a single, cylindrical membrane domain embedded in a large, homogenous two-dimensional fluid, e.g. a single-component phospholipid bilayer.

Today, there is a growing body of evidence that additional parameters such as molecular crowding and protein size should also be taken into account. These studies include molecular dynamics simulations^9^ and experiments in artificial membrane systems^10^ as well as heterologous expression systems^11^. While the models for diffusion of transmembrane proteins are well established, it is not straightforward to apply them to lipid-anchored proteins. Due to the small size of the GPI-anchor, which is well within the same order of magnitude as the constituents of the membrane (the lipids), a hydrodynamic model that describes diffusion of proteins with transmembrane domains, does not necessarily apply. In addition, the membrane part of GPI-anchored proteins only interdigitates with one half of the bilayer. This raised the question whether the frictional coupling between the membrane and the anchor also dominates diffusion of these proteins or if the ectodomain might have a significant influence. So far, diffusion studies of GPI anchored proteins in model membranes as well as in live cells have yielded contradictory results. While some studies claim that the size of the ectodomain is crucial (e.g.^12^,^13^,^14^), others propose the opposite (e.g.^15^,^16^). To clarify this contradiction, we have devised a comparative experimental scheme that examines diffusion of GPI-proteins *in vivo* on living cells, *in vitro* on supported membranes and *in silico* using computer simulations.

We exploit the unique advantages of African trypanosomes as a biological model. Although GPI-proteins fulfil essential functions on virtually all eukaryotic cell surfaces, they were discovered in these unicellular parasites, due to their shear abundance. In trypanosomes, a single type of GPI-anchored variant surface glycoprotein (VSG) covers the whole cell surface^17^, thereby effectively shielding the plasma membrane from recognition by the host immune system. The trypanosome genome contains hundreds of VSG genes, all encoding structurally similar, albeit immunologically distinct proteins. At any given time, the parasite expresses just one type of VSG. The mammalian host’s immune system responds with production of VSG-specific antibodies and eliminates the parasite population almost completely. Randomly occurring switches in the monoallelic expression of VSG genes, however, allow a subpopulation of trypanosomes to escape immune destruction by exposing a different VSG-coat, which is not detected by the previous antibody response. Thus, there is a strong selective pressure on the parasite VSG repertoire: VSGs have to be sufficiently similar to maintain the shielding function on the cell surface, e.g. during antigen switching, and they have to be sufficiently different to provide the molecular basis for antigenic variation^18^.

This unusual homogeneity of the trypanosome surface coat makes the VSG an ideal tool for studies on the impact of the protein’s soluble domain on lateral diffusion. Here, we specifically ask two questions: (A) to what extent does the axis length of the VSG influence its diffusion properties and (B) do VSG dimensions correlate with potentially diffusion-limited biological functions ? We decided to tackle these questions in a sequential experimental approach that involves stepwise abstraction from the natural state.

## Results

### Manipulation of VSG size on living trypanosomes

**Figure 1:**
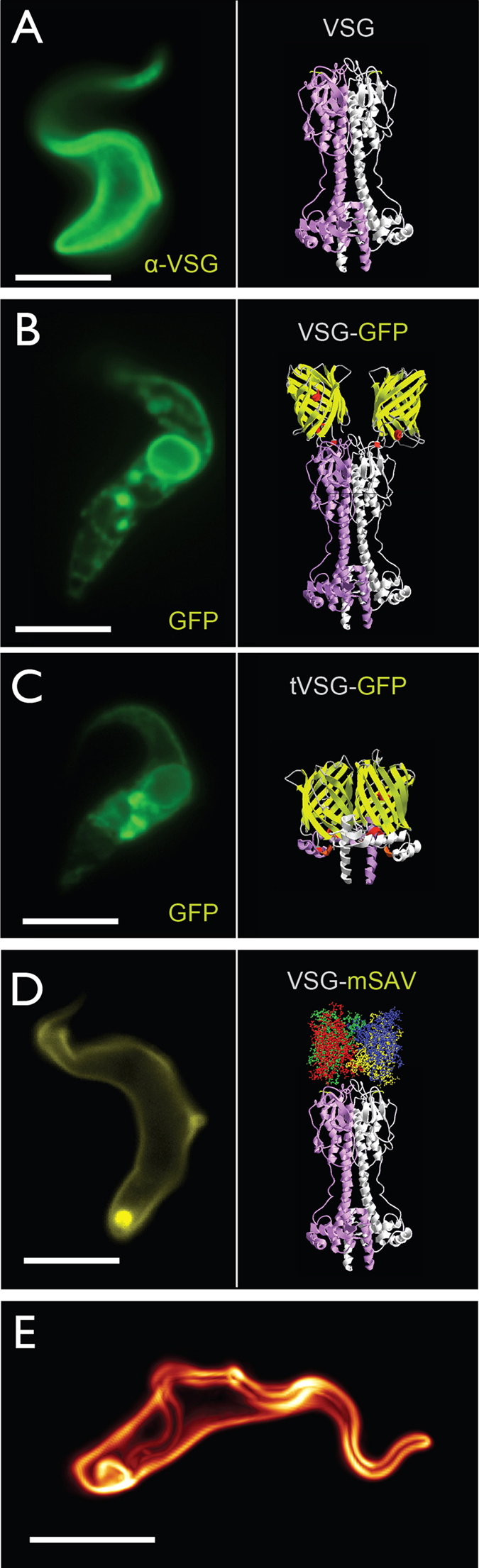
**Manipulation of the size of VSG by expression of fusion proteins and chemical labelling. a**) The VSG coat of chemically fixed bloodstream form trypanosomes is recognized by VSG-specific primary antibodies and green fluorescent secondary antibodies. **b**) Fusion of the green fluorescent protein (GFP) to the N-terminus of VSG M1.2 produces an enlarged VSG chimera (VSG-GFP; right). The transgenic trypanosomes reveal a strong green fluorescence, which, however, is restricted to the biosynthetic and endosomal compartments. No cell surface fluorescence is detected (left). **c**) Fusion of the C-terminal domain of VSG M1.2 to GFP (tVSG-GFP). From structural homology modelling (right) the resulting chimeric protein is expected to be significantly smaller than the native VSG. The fusion protein is expressed, however, it is also retained intracellularly (left). **d**) Live cell labelling of biotinylated trypanosomes with Alexa 488-conjugated streptavidin (left). The streptavidin molecule enlarges the VSG by 5 × 5.5 nm (right) **e)** Live cell labelling of trypanosomes with sulfo-NHS-Atto488. The small, membrane-impermeable fluorophore evenly tags the VSG coat of living trypanosomes. Scale bars: 3 μm.

Based on available structural information we generated differently sized GFP-fusion proteins of the VSG. The GFP was linked to the N-terminus of full size and truncated VSGs, in a position that had previously been shown to accept smaller peptide tags^19^. The fusion cassettes were integrated just upstream of the expression site-resident native VSG M1.2 gene, using the pKD gene expression system^19^. Three independent clones each were analysed for transgene expression. Although all cells revealed significant fluorescence, the signals of both fusion proteins were restricted to intracellular membranes and were absent from the cell surface (Fig. 1). Therefore we established a procedure for direct manipulation of VSG M1.6 on living parasites. Using sulfo-NHS-chemistry, the trypanosomes were dually tagged with biotin and the fluorescent dye ATTO-488 in a molar ratio of 2:1. This procedure quantitatively labelled the VSG-coat with both small tags (Fig. 1)^20^. The cells were transiently immobilised in a custom-made observation chamber, and diffusion coefficient and mobile fraction of the control VSG were determined by FRAP analysis (Table 1). Next, the VSG size was increased by addition of monovalent streptavidin (mSAV)^21^. The single femtomolar biotin-binding site of mSAV guaranteed that the VSGs on living cells remained tagged throughout the experiment. Based on the molecular structure of VSGs and their lateral spacing within the surface coat, we expected a 1:1 ratio of VSG and mSAV, which would result in an evenly enlarged VSG surface coat. Monovalent streptavidin was used, as tetravalent SAV cross-linked the biotinylated VSGs on the cell surface and caused rapid cell death. FRAP revealed that the fraction of VSG-mSAV molecules freely moving in the plane of the plasma membrane, in the following referred to as the mobile fraction, was around 75%, which is comparable to that of wild type VSG. The diffusion coefficient of VSG-mSAV, however, was reduced three-fold (Table 1). Thus, an increase of VSG axis length by one-third has a marked influence on the diffusion of the protein on the cell surface of trypanosomes.

For reduction of VSG size on living cells we exploited the fact that VSGs are differentially sensitive to trypsin cleavage^22^. The VSG M1.6 used in this experiment is specifically cleaved at the hinge-region between the N- and C-terminal domains, which reduces the size of the membrane-anchored protein to about 20% of the full VSG. Exponentially growing trypanosomes expressing VSG M1.6 were harvested and, after labelling of the cell surface using sulfo-NHS ATTO-488, treated with saturating amounts of trypsin at 37 °C for 5 min. Following protease incubation, parasites were washed three-times and immobilised for FRAP analysis. The mobile fraction of trypsin-treated VSGs was about 75%, i.e. comparable to both that of wild type protein and enlarged VSG-mSAV. The diffusion coefficient of trypsinized VSG, however, was almost twice as large as that of native VSG and more than 4-fold larger than VSG-mSAV (Table 1). Thus, the *in vivo* experiments are compatible with a model in which VSG size significantly influences its diffusion coefficient, while leaving the mobile fraction unaffected.

### Manipulation of VSG size in supported membranes

The results obtained with living trypanosomes were challenged in a defined system of artificial supported membranes. VSG M1.6 was purified in its native, GPI-anchored membrane form from large-scale trypanosome cultures^23^. The isolated protein was biotinylated and fluorescently labelled with sulfo-NHS-biotin and sulfo-NHS-ATTO 488, respectively, and incorporated into supported membranes, which had been prepared by spreading small unilamellar lipid vesicles onto hydrophilic glass substrates. The lateral density of VSG on the supported membranes was adjusted to 5500 proteins per μm^2^(free average distance d = 7 nm), i.e. the concentration of proteins was about 15% of that found in the VSG-coat. At this concentration the diffusion of VSG is not impaired by lateral protein-protein interactions. VSG diffusion coefficients and mobile fractions were determined by FRAP analysis and found to be within the expected range.

**Figure 2:**
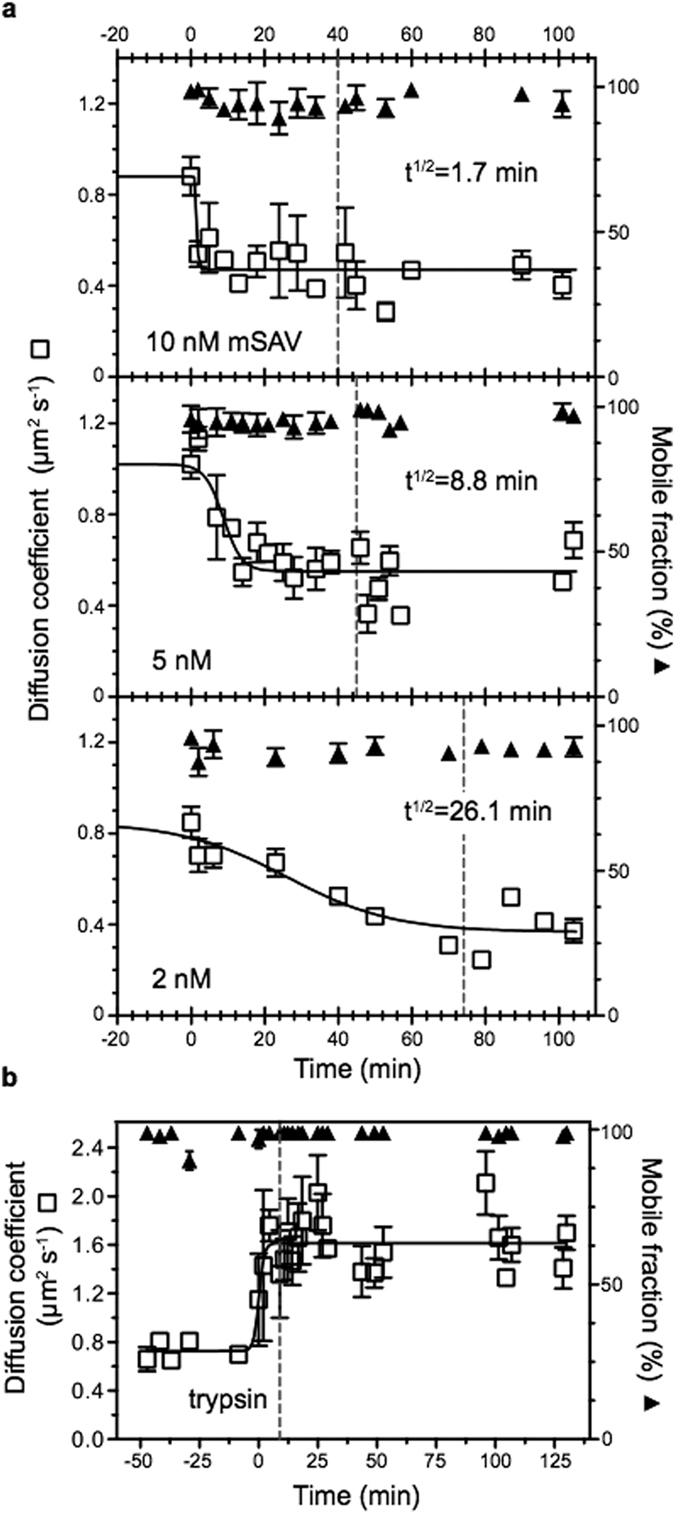
**The diffusion coefficient depends on protein size. a**) Enlargement of biotinylated VSG by binding of monovalent streptavidin (mSAV) affects the diffusion coefficient (□), but does not influence the mobile fraction (▲). VSG was incorporated into supported membranes and FRAP measurements were conducted before and after mSAV was added at t_0_. Depending on the concentration of mSAV (10, 5 and 2 nM), the diffusion coefficients were reduced with different kinetics. Fitting the data to a curve with variable slope (full line) yielded the half-life values (t^1/2^) for the reduction of the diffusion coefficients. The dotted line marks the time of removal of unbound mSAV by washing. Data are mean values ± SD. **b**) Size reduction of VSG by trypsin cleavage yields a larger diffusion coefficient (□) without impacting on the mobile fraction (▲). After addition of trypsin at t_0_ the diffusion coefficient doubles within 2 min. The dotted line marks the removal of trypsin by washing. Data were fitted to a curve with variable slope (full line) and are mean values ± SD.

**Figure 3:**
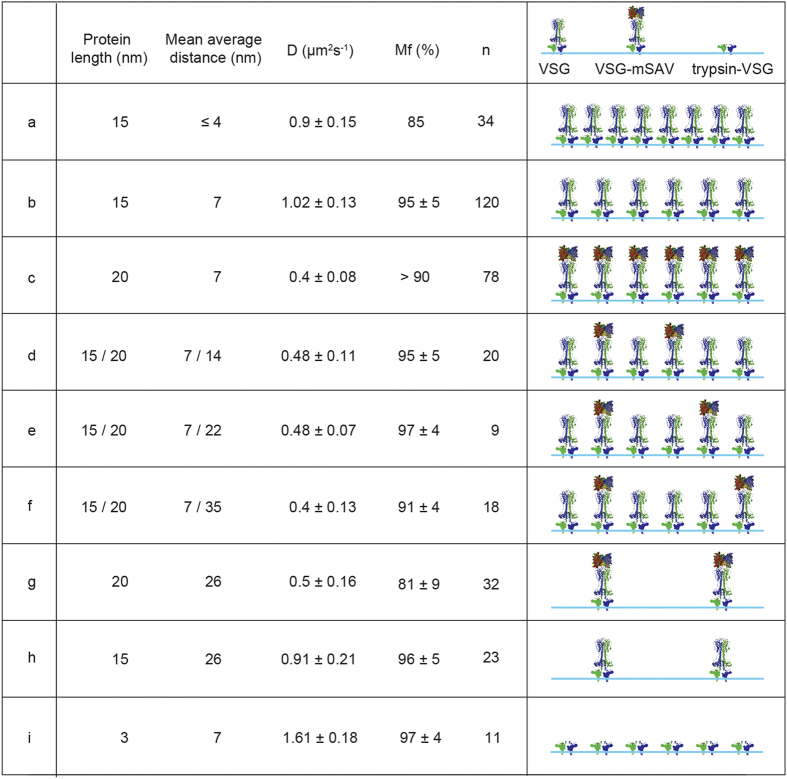
Influence of protein size and density on diffusion coefficients and mobile fractions in supported membranes. The combination of table and schematic illustration summarizes the results from various experiments. **a**) Diffusion of native VSG at VSG coat density. **b**) An artificial coat with more than double mean average distance between native VSG molecules. **c**) The same density as in b, but with mSAV-tagged VSG. **d**) mSAV-tagged VSG diluted 1:2 with native VSG. **e**) VSG-mSAV diluted 1:4 with native VSG. **f**) VSG-mSAV diluted 1:8 with native VSG. **g**) VSG-mSAV diluted 5-fold compared to c. **h**) Native VSG diluted 1:5 compared to b. **i**) Trypsin-treated VSG coat with same density as in b. All data are means ± SD.

**Figure 4:**
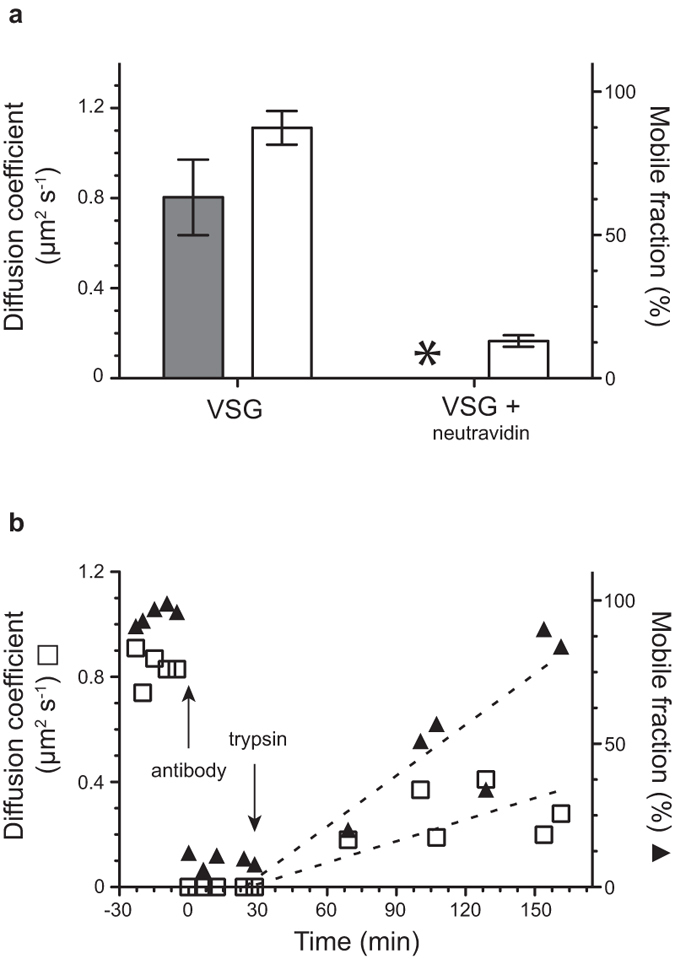
**Cross-linking of VSG in supported membranes. a**) As a control for the mSAV experiment, tetravalent neutravidin was added to biotinylated VSG. This leads to rapid crosslinking and immobilization of the protein complexes. The mobile fraction 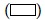 was reduced 7-fold and no diffusion coefficient 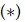 could be calculated using Soumpasis diffusion algorithm for complexed proteins 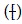. The diffusion coefficient 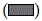
and mobile fraction 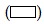
of untreated VSG was normal. Data are means ± SD. **b**) Treatment of VSG in supported membranes with a VSG-specific antibody causes immediate immobilization of the protein complexes that is slowly reversed after trypsin digestion. Arrows mark the addition of rabbit anti-VSG M1.6 antibody and trypsin, respectively. Diffusion coefficient (□), mobile fraction 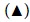. The dotted lines represent linear regression and are guidelines for the eye.

In the next step, mSAV was added onto the supported VSG-membranes in final concentrations of 2, 5 and 10 nM, respectively. The mobile fraction of VSG did not change after addition of mSAV, which is in accordance with the observations made with living cells. The diffusion coefficient, however, was reduced two-fold, which is also in agreement with the results obtained with living cells. While in the presence of 10 nM mSAV the diffusion coefficient decreased to 0.4 ± 0.15 μm^2^ s^−1^ with a half-life of 1.7 min, incubation with 5 nM and 2 nM mSAV resulted in longer half-lives of 8.8 and 26 min, respectively (Fig. 2A, Fig. 3, panels b and c). The experiments were tested for non-specific binding of mSAV to non-biotinylated VSG by washing the supported membranes, and no significant changes in diffusion coefficients or mobile fractions were observed. Furthermore, neutravidin was used as a control for the monovalent binding properties of mSAV. The addition of tetravalent neutravidin led to an almost instantaneous breakdown of VSG mobility (Fig. 4). This result is in agreement with the cross-linking activity of SAV observed on living trypanosomes. Likewise, the addition of anti-VSG M1.6 antiserum resulted in immediate cessation of VSG mobility in supported membranes. This was fully reversible by the addition of trypsin, which released the immunoglobulins (Fig. 4B).

Having shown that an increase in VSG size led to a decrease in its diffusion coefficient, we asked if this was caused by augmented protein-protein interactions between the elongated VSG. To address this question we prepared supported membranes with native and labelled VSGs in different molar ratios, resulting in a range of different distances between individual biotinylated VSG molecules, without altering the overall VSG density (Fig. 3, panel b). The dilution of mSAV-tagged VSG with native, non-labelled protein led to a stepwise increase in the mean average distance between VSG-mSAV to 14, 22 and 35 nm (Fig. 3 panels d-f). The measurements revealed that the diffusion coefficient of VSG-mSAV was not influenced when diluted with native VSG. Thus, the smaller diffusion coefficient of the enlarged VSG was not due to stronger lateral protein-protein interactions. This result was confirmed by 5-fold overall dilution of the artificial VSG-coat. We prepared samples of biotinylated VSGs on supported membranes with 1000 proteins per μm^2^, which corresponds to a mean average protein distance of about 26 nm. As shown in Fig. 3 (panels g and h), the diffusion coefficients of diluted native VSG and VSG-mSAV were comparable to those measured in concentrated artificial coats. This underlines that protein size alone, independent of lateral density and potential physicochemical interactions, can determine the diffusion coefficient of GPI-proteins.

The above results predict that a decrease in protein size should increase the diffusion coefficient of VSG in supported membranes. To test this, VSG labelled with sulfo-NHS ATTO-488 was incorporated into supported membranes and treated with trypsin for 10 min, followed by washing to remove the protease along with VSG cleavage products. The efficiency of trypsin digestion was monitored by the gradual reduction of the overall fluorescence signal to a steady-state level. The diffusion coefficients and mobile fractions of VSG were determined before, during and after protease treatment. The mobile fraction remained unaffected, whereas the diffusion coefficient increased very rapidly from 1.02 +/−0.13 μm^2^s^−1^ to 1.61 +/−0.18 μm^2^s^−1^, which means that the C-terminal part of the VSG diffuses almost twice as fast as the full-sized protein (Figs. 2B and 3, panel i). This result further supports the conclusion that the diffusion coefficient of GPI-anchored proteins, but not the mobile fraction is determined by protein size.

### Simulation of VSG diffusion

**Figure 5:**
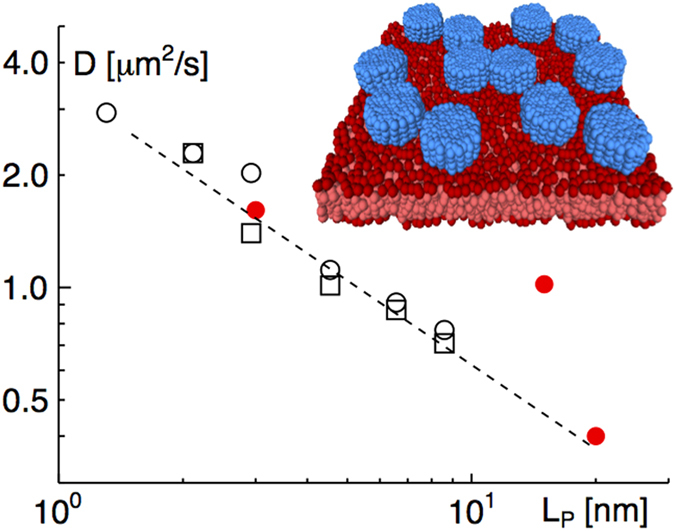
Influence of protein size on diffusion coefficients in simulations. Simulations of membrane protein diffusion at protein densities of 3100 μ m^-2^ and 6200 μ m^-2^ (open circles and squares, respectively) reveal a distinct reduction of the diffusion coefficient with varying lengths of the protein soluble domain, L_P_. Error bars are smaller than symbol size. Empirically, the gross tendency is well described by a scaling 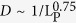 (dashed line). Simulation data are in agreement with the corresponding experimental data for VSG proteins at 9 nm spacing (red filled circles). Inset: Snapshot of the simulation. For better visibility water beads are not shown, and periodic images of simulation boxes are displayed.

In a further step of abstraction from the natural state we asked whether simulations could quantitatively reproduce and explain our experimental findings on VSG diffusion. To this end, we employed a coarse-grained simulation approach that is frequently used to study fully hydrated membrane systems on scales larger than 1 nm. Lipids and VSG proteins were structurally simplified to allow for large spatial and temporal scales to be covered by our simulations (see Fig. 5, inset for a representative snapshot of our simulation). From the time course of the simulation, we were able to determine the diffusion coefficient of VSG-like model proteins in dependence on the length of the soluble domain, L_P_, and with respect to the overall protein density on the membrane. We found that varying the protein density in the range of the experimental values had no significant effect whereas a change in the length, L_P_, of the soluble domain strongly affected the diffusion coefficient of the protein (Fig. 5). These simulation results are in favourable agreement with our experimental data (Fig. 2). Moreover, both data sets follow an empirical scaling of the diffusion coefficient 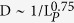, which highlights that the soluble domain has a significant influence on the diffusion that goes far beyond the Saffman-Delbrück relation.

## Discussion

The trypanosome surface coat is well suited for studies on the diffusion of GPI-anchored proteins, as it basically consists of a single type of protein, the VSG. Evolution has moulded VSGs for collective functionality. They adopt very similar molecular shapes and dimensions, because all members of this large protein family must be able to form a switchable and extraordinarily dense protein layer that shields invariant membrane proteins such as receptors and channels. No host antibody can penetrate this VGS layer^24^, as the distance between individual VSGs is less than 4 nm. In addition to this remarkable molecular crowding, VSGs need to be evenly distributed on the cell surface, which means they must diffuse freely. In fact, we found that VSG mobility is high, even at lateral densities resembling the surface coat concentration.

The results of the present study indicate that the VSG diffusion coefficient is inversely related to its molecular axis length, whereas the mobile fraction is not affected by protein size. The hydrodynamic Saffmann-Delbrück model cannot faithfully predict the effect of the size of the ectodomain on diffusion of GPI-anchored proteins, as we demonstrate that the extracellular protein part of GPI-proteins contributes significantly to their diffusion: larger GPI-proteins diffuse more slowly than smaller ones.

**Figure 6:**
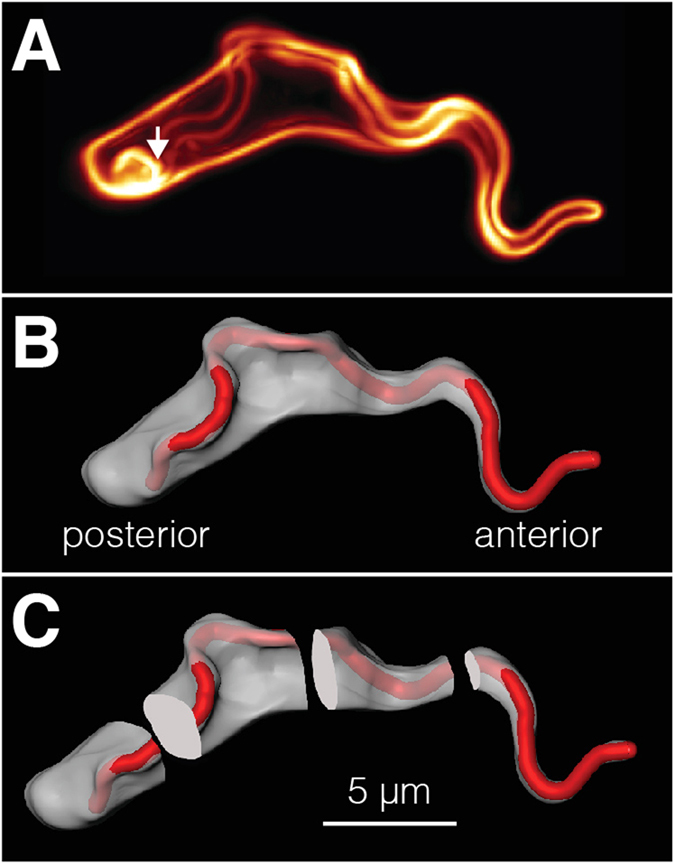
**Determination of trypanosome cell surface areas. a**) The measurement of surface areas was done using high-resolution 3D fluorescence microscopy as described^19^. The arrow indicates the position of the flagellar pocket. **b**) Based on the 3D-data set a volume model was rendered using the AMIRA software package (FEI). Red colour indicates the flagellar surface and the cell body membrane is displayed in grey. **c**) The trypanosome surface model was digitally divided into four sections of 5 μm length each and the surface areas of these sections were calculated individually.

We tentatively propose a model in which the efficiency of trypanosome surface coat recycling is linked to the VSG’s diffusion coefficient, which in turn is tuned by its molecular axis length. The VSG coat of trypanosomes is continuously and rapidly internalized and recycled through a tiny surface area at the posterior cell pole, the flagellar pocket^19^ (Fig. 6). The restriction of endo- and exocytosis to the flagellar pocket has implications for VSG coat dynamics^17^. The scenario in fact is reminiscent of the ‘narrow escape problem’ in which particles are considered diffusing on a given surface to reach a small predefined opening^25^,^26^. When reappearing on the cell surface, the VSG molecules have to be efficiently randomized. Otherwise, a trafficking ‘short-circuit’ would be generated, in which exocytosed VSG would preferentially be re-endocytosed, while VSGs located at the anterior end of the cell would hardly enter the endosome. The need for VSG randomization becomes especially important in the course of antigenic variation, when a new VSG coat replaces the old one. Therefore, we asked whether the measured diffusion coefficients allow for an efficient randomization of VSG and made the following estimate. The distance between the flagellar pocket and the anterior pole of the cell is about 15 μm. One surface coat equivalent is endocytosed every 10 min^19^. Within this period the mean travel distance r(t) = (4Dt)^1/2^ of VSG (D = 0.03 μm^2^ s^−1^) is about 9 μm, while membrane lipids travel around 23 μm (D = 0.22 μm^2^ s^−1^;^27^). Thus, VSG randomization on the cell surface is a diffusion-limited process, which operates within the range of the real cellular distances. Interestingly, the trypanosome cell architecture also contributes to efficient VSG randomization. Using 3D-fluorescence microscopy, we have measured the cell surface of the spindle-shaped trypanosomes^20^ and divided it into four 5 μm-sections along the longitudinal axis of the cell (Fig. 6). The whole pellicular surface coat consists of 100 μm^2^ of membrane, of which 18 μm^2^ are located at the most posterior section, 22 μm^2^ and 20 μm^2^ are found in the two middle sections and just 13 μm^2^ belong to the most anterior part of the cell. This means that more than 80% of all VSG molecules are within reach of the calculated mean travel distance of 9 μm, which should guarantee a rather efficient randomization of recycled VSGs. We found that an increase in VSG size by one-third leads to a three-fold smaller diffusion coefficient. This difference would have a marked impact on the mean travel distance of VSG, which decreases to 4.9 μm / 10 min. Thus, if VSG size increases by 1/3, almost half of the surface coat (46%) would be no longer within reach of a 10-minute mean travel distance. In contrast, downsizing the VSG would not markedly improve VSG randomization. While the reduction of VSG size by trypsin treatment results in an increase of the diffusion coefficient (D = 0.043 μm^2^ s^−1^), the mean travel distance only rises to 9.8 μm / 10 min.

We have shown that antibody-complexed VSGs move directionally towards the flagellar pocket, driven by hydrodynamic drag generated by cell motion^18^. As antibody clearance is an active process that operates at least an order of magnitude faster than diffusion it is not relevant for the general process of VSG randomization discussed here.

We speculate that the evolutionarily conserved dimension of VSGs is, at least in part, a function of its diffusion coefficient, which has to be maintained within a range that enables functional VSG coat randomization and hence, efficient coat recycling and switching. This would be a remarkable example for protein diffusion acting as an organizing principle on a cellular membrane.

In contrast to the trypanosome surface coat, the plasma membrane of most other cells is decorated with an unknown number of proteins of varying sizes and physical properties. Thus, it will prove difficult to pinpoint the role of diffusion for the function of individual membrane proteins. Interestingly, however, many pathogens feature a homogenous surface coat consisting of proteins with remarkably similar molecule axis length. The VSG surface coat of African trypanosomes is 15 nm thick. The only distantly related South American species, *Trypanosoma cruzi,* features a 15 nm thick GPI-anchored mucin coat. Likewise, the plasma membrane of extracellular merozoites of the malaria parasite *Plasmodium* is covered with a 15-20 nm thick surface coat consisting of GPI-anchored proteins. The infective yeast-like fungus *Pneumocystis jirovecii* undergoes antigenic variation of GPI-anchored major surface glycoproteins that form a 20 nm surface coat. The free-living protist *Paramecium tetraurelia* features a variable GPI-anchored surface coat that is also 17 - 22 nm thick. Similarly the variant surface protein (VSP) coat of *Giardia lamblia* is 18 nm thick and uniform.

Therefore, we hypothesize that the need for diffusion-limited randomization of mostly GPI-anchored membrane proteins might explain the surprisingly similar dimensions of the surface coats of various cells that undergo antigenic variation. Thus, the strategy of controlling protein diffusion by molecular size must not necessarily be a peculiarity of African trypanosomes.

## Methods

### Preparation of monovalent streptavidin

Monovalent streptavidin was made using a protocol by Howarth *et al.* 2006^21^. Briefly, biotin-binding (alive) and non-binding (dead) streptavidin monomers were expressed separately in *E. coli* strain Rosetta-BL21-(DE3)-pLysS using the plasmids pET21a-streptavidin-dead (Addgen: 20859) and pET21a-streptavidin-alive (Addgene: 20860). After cell extraction, proteins in inclusion bodies were washed, denatured in guanidine-HCl, mixed in 1:4 (alive:dead) molar ratio, refolded in PBS, precipitated in ammonia-sulfate and finally purified by affinity chromatography on nickel-NTA agarose (QIAGEN, Hilden). The monovalent streptavidin (mSAV) was dialyzed against PBS. Fluorescence labelling of mSAV was achieved with sulfo-NHS ATTO-488 (ATTO-TEC, Siegen) following the manufacture’s instructions. The unbound dye was removed by PD-10 gel-filtration (GE Healthcare, Solingen). mSAV was eluted in TNC-buffer (20 mM Tris-HCl, pH 7.4, 50 mM NaCl and 0.5 mM CaCl2) and stored at −20 °C.

### Trypanosomes

*Trypanosoma b. brucei* strain Lister 427 MITat 1.6 (M1.6) bloodstream form expressing VSG M1.6 was cultivated in HMI-9 medium, supplemented with 10% foetal calf serum, at 37 °C and 5% CO_2_. Unless otherwise stated, cell density was kept below 5 × 10^5^ cells/ml to ensure analyses at the exponential phase of cell growth.

### Cell surface labelling

10^7^ trypanosomes were washed three times in ice-cold trypanosome dilution buffer (TDB; 5 mM KCl, 80 mM NaCl, 1 mM MgSO_4_, 20 mM Na_2_HPO_4_, 2 mM NaH_2_PO_4_, 20 mM glucose, pH 7.6), resuspended at 1 × 10^8^ cells/ml and incubated in the presence of 20 μM EZ sulfo-NHS-biotin (Thermo Scientific, Sunnyvale) and/or 10 μM sulfo-NHS ATTO-488 for 15 min on ice in the dark. Unbound biotin and/or fluorescent dye were removed by three washes with ice-cold TDB.

### Binding of mSAV on living cells

All experiments were performed on ice. 10^7^ biotinylated and fluorescently labelled trypanosomes were incubated with 0.2 × 10^−9^ moles mSAV for 30 min. After incubation the cells were washed three-times to remove unbound mSAV.

### Immobilization of trypanosomes

10^7^ trypanosomes were resuspended in 20 μl TDB. 3 μl of the cell suspension was mixed with 5 μl of 10% (w/v) type-A gelatine in TDB (from porcine skin; Sigma-Aldrich) and pipetted between two cover slips. The coverslips were mounted in a temperature-controlled microscope sample holder. The experimental conditions were optimised for transient immobilisation of trypanosomes at 20 °C. At this temperature the cells are metabolically largely unaffected and trapping of the parasites up to at least 3 hours in gelatine does not impair their viability. On release from gelatine immobilization cells proliferated normally.

### Trypsin-cleavage of VSG on living cells

Fluorescently labelled (sulfo-NHS ATTO-488) trypanosomes were suspended in 1 ml TDB; 10 μg trypsin 250 (Becton Dickinson, Heidelberg) was added and the cells were incubated for 5 min at 37 °C, followed by 3 washes with TDB. The viability of the parasites was microscopically monitored throughout the experiments.

### Purification of membrane form VSG

Membrane form VSG (mfVSG) was purified as reported^23^. After purification of mfVSG by HPLC, proteins were biotinylated with EZ sulfo-NHS-biotin and fluorescently labelled with sulfo-NHS ATTO-488. Unbound biotin and dye were washed away and mfVSG was suspended in TNC-buffer and stored at −20 °C. The degree of labelling (typically 2 for sulfo-NHS ATTO-488) was determined following the manufacture’s instructions.

### Incorporation of mfVSG into supported membranes

Cover slips (Hecht Assistant, No. 1, 24 mm diameter) were consecutively cleaned by sonication in acetone, ethanol and methanol. After rinsing with deionized water the cover slips were immersed in a mixture of 1:1:5 (v/v/v) 30% ammonia : 30% hydrogen peroxide : dd-H_2_0 for 30 min at 60 °C, followed by intensive washing with deionized water. The cover slips were dried at 70 °C, stored in a vacuum desiccator and used within 2 days. Supported membranes were prepared by the fusion of small unilamellar vesicles (SUV) to the hydrophilic glass slides. SUVs were prepared from 1-stearoyl-2-oleoyl-sn-glycero-3-phosphocholine (SOPC, Avanti Polar Lipids, Inc., Alabama). For this, an appropriate volume of SOPC in chloroform was dried in a nitrogen gas stream and rehydrated in TNC-buffer under vigorous shaking. The final lipid concentration was 1 mM. SUVs were made by sonication of the SOPC solution until the suspension became translucent. The hydrophilic coverslips were mounted in a sample chamber and typically 1 ml of the SUVs was applied and incubated for 30 min at 37 °C. The supported membranes were washed intensively with TNC-buffer and 0.2 nM biotinylated and/or fluorescently labelled mfVSG was added to the buffer reservoir above the supported membranes. The samples were then incubated for 30 min at 37 °C. Non-incorporated mfVSG was removed by intensive washing of the supported membranes with TNC-buffer.

### Determination of lateral protein-protein distances on supported membranes

Supported bilayers were produced as described above and 0.2 nmol mfVSG in TNC-buffer was added to the buffer reservoir. Samples were taken immediately (S_1_) and after 30 min (S_30_). Protein concentrations of these samples were determined by immuno-detection and near infrared fluorescence scanning of dot-blots. The differences between samples S_1_ and S_30_ were normalized to the total amount of incorporated mfVSG on supported membranes. A linear dilution series of VSG served as a standard. VSG dimers have a lateral size of 6.5 × 4.5 nm and can rotate freely in fluid membranes^20^. The resulting effective radius r_VSG_ is 4 nm. In order to calculate the free distance, d, between VSG dimers at various concentrations, c, we assumed a hexagonal protein distribution. Thus, d = 2*(sqrt(1/(c*pi))- r_VSG_).

### Cleavage of VSG by trypsin on supported membranes

Proteolysis was done in TNC-buffer containing 0.06% (w/v) of trypsin. After 10 min the samples were washed with trypsin-free TNC-buffer to remove the protease and protein fragments.

### Binding of mSAV to mfVSG on supported membranes

Binding of mSAV (preparation see above) or neutravidin (Invitrogen, Darmstadt) to biotinylated mfVSG was done by pipetting a five-fold molar excess of avidin to the buffer reservoir of the supported membranes. Samples were analysed immediately for time-resolved FRAP-experiments or were incubated for 30 min at 37 °C in the dark before analysis.

### Microscopy and FPAP-measurements

An inverted fully automated wide-field microscope (TILL Photonics, Gräfelfing) was equipped with a Polychrome V (TILL Photonics), a CCD camera (Sensicam, pixel size 6.45 μm, PCO, Kelheim), a Yanus digital scan head, 473 nm and 561 nm (Cobolt Inc., Solna) lasers, and the corresponding emission and excitation filter cubes. Fluorescence recovery after photo-bleaching (FRAP) experiments were done with a Nikon 60x objective lens (NA 1.45). Unless otherwise stated all FRAP-experiment were performed with sulfo-NHS ATTO-488 labelled proteins. Maximum laser power was used for irreversible photo-bleaching of the region of interest. The polychrome V was used as a light source to record pre- and post-bleach frames with an excitation wavelength of 488 nm. Fluorescence emission was filtered using a BrightLine full multiband 488/561 filter set (Semrock). All equipment was controlled with the “Live acquisition” software (TILL Photonics) and images were analysed with the Offline Analysis software packages (TILL Photonics). Protein diffusion was quantified by fluorescence recovery after photo-bleaching (FRAP) measurements. Diffusion on living trypanosomes was analysed by line-FRAP. Data evaluation was done according to Phair *et al*.^28^. Circular regions of interest were bleached on supported membranes and diffusion was analysed according to Soumpasis *et al*.^29^. All FRAP-experiments were performed at 20 °C.

### Simulations

For all membrane simulations we used dissipative particle dynamics (DPD) that is frequently used to perform simulations beyond the limitations of molecular dynamics. Diffusion properties of membrane proteins have been assessed previously with the aid of DPD^8^,^30^,^31^. For technical details of the simulation (equations of motion, integration schemes, definition of parameters and potentials) we refer the reader to an extensive introduction in Ref.^32^. In brief, we relied on previously reported parameters, i.e. the soft-core radius of all simulation beads was r_0_ = 1 and the simulation time step was D_t_ = 0.01; dissipation and noise of the thermostat were set to 3 and 4.5, respectively. Repulsion parameters between individual beads (reflecting the degree of hydrophobicity) were set to a_WW_ = a_WH_ = a_HH_ = a_TT_ = 25, a_WT_ = a_HT_ = 200; here the indices denote water (W), lipid head group (H), and lipid tail group (T). Lipids were taken as linear polymers HT_*3*_, connected by Hookean springs (spring constant *k* = 100, relaxation distance *l*_*0*_ = 0.45) and equipped with a bending rigidity (bending constant *k* = 10). VSG proteins were modelled as cylinders with a radius 2.75r_0_ and length L_P_. Membrane association was assured by two lipid-like HT_*3*_groups attached to the bottom of the cylinder in a distance 1.35r_0_ from the cylinder’s symmetry axis. Each cylinder consisted of 103 chains of H beads, H_*n*_, with *n* *=* 2, 4, 6, 10, 15, 20 determining L_P_. Beads were connected to their next neighbours via Hookean springs in each circular cross-section of the cylinder and along each chain H_*n*_; rigidity of the protein was achieved by using a bending stiffness for each H_*n*_ chain (parameters for springs and bending as before). The density of water beads and lipids was set to 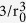 and 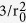, respectively. The size of the membrane patches with area L × L was L = 20r_0_, the adjacent water layer was chosen to be at least L_P_ + 10r_0_.

In each run, we equilibrated the system for 50,000 time steps to a tensionless state using a barostat, then we fixed the equilibrated system size and monitored the proteins’ motion for 10^8^ time steps D_t_. Diffusion coefficients were extracted from these time series as described earlier^31^. For comparison with experimental data, we converted simulation units via r_0_ ↔ 0.9 nm (yielding a lipid bilayer thickness of 3.5-4.5 nm) and D_t_ ↔ 40 ps (by comparison of the diffusion of individual lipids with experimental data).

## Additional Information

**How to cite this article**: Hartel, A. J. W. *et al*. The molecular size of the extra-membrane domain influences the diffusion of the GPI-anchored VSG on the trypanosome plasma membrane. *Sci. Rep.* **5**, 10394; doi: 10.1038/srep10394 (2015).

## Figures and Tables

**Table 1:** Diffusion properties of VSG on the cell surface of living trypanosomes.

	**D (μm^2^s^−1^)**	**Mf (%)**	**n**
native VSG	0.027 ± 0.008	80 ± 5	20
VSG-mSAV	0.010 ± 0.003	75 ± 12	17
trypsin-VSG	0.043 ± 0.013	76 ± 5	18

(D) Diffusion coefficient; (Mf) mobile fraction. Data shown as mean ± SD.
